# Case Report: Unveiling the role of genetic abnormalities in myelomatous pleural effusion: a case of IgG lambda-type multiple myeloma

**DOI:** 10.3389/fonc.2025.1554956

**Published:** 2025-06-20

**Authors:** Shuangyan Chen, Lanfang Yan, Yinjuan Mo, Daoyin Zhou, Yan Chen

**Affiliations:** ^1^ Department of Laboratory, The First People’s Hospital of Shuangliu District, Chengdu, Sichuan, China; ^2^ Department of Laboratory, Zhujiang Hospital, Southern Medical University, Guangzhou, Guangdong, China; ^3^ Department of Experimental Diagnostics, Shanghai Changhai Hospital, Shanghai, China

**Keywords:** multiple myeloma, myelomatous pleural effusion, integrated treatment, diagnostic methods, chemotherapy regimen

## Abstract

A 73-year-old male with a history of multiple myeloma presented with progressively worsening left shoulder pain that was unresponsive to conservative management. Following comprehensive diagnostic procedures, including imaging and genetic testing, he was diagnosed with IgG lambda-type multiple myeloma, complicated by the development of myelomatous pleural effusion (MPE). The patient’s treatment regimen consisted of the PDD chemotherapy combination, including Bortezomib, Doxorubicin, and Dexamethasone, alongside adjunctive therapies aimed at controlling the pleural effusion. Post-treatment evaluation revealed resolution of the pleural effusion, with no detectable myeloma cells in the pleural fluid. This case illustrates the clinical utility of combining chemotherapy with supportive treatments for the effective management of MPE in multiple myeloma patients. The patient’s positive response highlights the significance of tailoring therapeutic approaches to the individual, emphasizing the role of personalized care in improving clinical outcomes in similar complex cases of MPE.

## Introduction

Multiple myeloma (MM) is a malignant hematologic disorder characterized by the aberrant proliferation of monoclonal plasma cells, primarily affecting the elderly population. Despite significant advances in therapeutic options, such as autologous stem cell transplantation, immunomodulatory drugs (IMiDs), and proteasome inhibitors (PIs), MM remains incurable, with patients at risk of relapse (1–3). MPE is a relatively rare complication within the treatment spectrum of MM, exacerbating patient conditions and complicating the selection of treatment plans and prognostic assessments. The incidence of MPE in MM patients ranges from 0.5% to 3.2%, often indicating disease progression and poor prognosis (4–6).

The diagnosis of MPE leverages the routine cytological examination of pleural effusion for its cost-effectiveness, convenience, speed, and high specificity, making it a crucial tool for enhancing the detection rate of malignant pleural effusions and providing an essential basis for stratified clinical diagnosis and treatment (1, 7, 8). Additionally, the application of molecular biological techniques such as fluorescent *in situ* hybridization (FISH) and flow cytometry deepens the molecular and cellular understanding of MM. These methods help identify specific genetic abnormalities and abnormal plasma cell populations, offering more precise treatment options for patients (9, 10). Notably, advancements in minimal residual disease (MRD) detection technologies provide new perspectives and strategies for monitoring and managing MM treatments (11–13).

While progress has been made in the treatment of MM, managing high-risk or relapsed/refractory MM patients remains a challenge (14–16). This study details the diagnosis and treatment of a 73-year-old male patient with MM, with a particular focus on the diagnosis and treatment of MPE. Employing a comprehensive array of diagnostic approaches—including complete blood counts, renal function tests, electrolyte and liver function assessments, thoracoabdominal CT, PET-CT scans, bone marrow cytology, flow cytometry, serum immunofixation electrophoresis, urine light chain analysis, and FISH technology—not only was MM and its rare complication MPE successfully diagnosed, but specific genetic abnormalities were also identified, providing a precise treatment plan for the patient. The patient underwent a series of treatments, including the PDD chemotherapy regimen, and effectively managed pleural effusion through intrathoracic injections, demonstrating the significant effects of integrated treatment in managing MM and its complications. This case emphasizes the importance of comprehensive diagnostic techniques in identifying and treating MM-related complications, offering valuable clinical experiences and methodological guidance for future diagnostics and treatment of MM and its complications, especially in handling rare complications like MPE. Moreover, this study highlights the crucial role of understanding the pathological mechanisms and genetic background of MM in developing personalized treatment plans.

## Materials and methods

### Cytological diagnosis of multiple myeloma: bone marrow and pleural fluid sample analysis

Bone marrow aspirate was collected from the patient through bone marrow puncture, and pleural effusion samples was obtained from patient thoracic drainage. Following collection, standard bone marrow smear techniques were employed to prepare slides for microscopy. Slides were fixed in methanol and stained for 15 minutes with Wright-Giemsa stain, then rinsed with water and air-dried to enhance the visibility of cellular structures. Different cell types were carefully observed and counted under a 1000x oil immersion microscope. For each sample, a hemocytometer was used to enumerate total and nucleated cell counts, calculating the percentages of lymphocytes, macrophages, neutrophils, and myeloma cells among nucleated cells. To ensure accuracy and repeatability, all counts were performed in triplicate and averaged.

### Flow cytometry analysis of bone marrow cell immunophenotype

Mononuclear cells were isolated from patient bone marrow samples using Ficoll-Paque PLUS (GE Healthcare) density gradient centrifugation. The isolated nucleated cells were resuspended in PBS buffer and adjusted to an appropriate cell concentration. Cells were incubated on ice for 30 minutes with fluorescently conjugated monoclonal antibodies to label specific cell surface markers, including CD45, CD38, CD138, CD19, and immunoglobulin light chains λ (cLambda) and κ (cKappa). Post-incubation, cells were analyzed on a BD FACSCanto™ flow cytometer. Fluorescence intensity for each antibody was measured, identifying nucleated cell populations via CD45-positive gating and calculating the percentages of lymphocyte and abnormal plasma cell populations. Data were processed and analyzed using FlowJo™, including gating strategies for cell populations, analysis of forward and side scatter properties, and expression levels of cell surface markers. Results were presented as the proportion of each cell population within the total nucleated cells and the characteristic expression patterns of abnormal plasma cells.

### Serum immunofixation electrophoresis

Patient serum samples were collected and allowed to clot at room temperature before centrifugation to obtain clear serum for electrophoresis analysis. Prepared serum samples were diluted appropriately and loaded onto precast gels. Electrophoresis was conducted on polyacrylamide gels using a constant current power supply to separate different immunoglobulin subtypes. Following electrophoresis, gels were stained with Coomassie Brilliant Blue to visualize protein bands. The gels were photographed using a Bio-Rad Gel Doc™ system, and protein bands were qualitatively analyzed using corresponding software.

## Case

A 73-year-old male patient reported persistent pain in the left shoulder for five months without a clear initiating cause, noting that the pain intensified with movement. During outpatient consultation, based on his symptoms and further examination findings, he was diagnosed with multiple myeloma and admitted for treatment 12 days after the diagnosis. Thoracic and abdominal CT scans revealed the presence of bilateral pleural effusion and ascites, along with multiple osteolytic lesions, which are characteristic manifestations of multiple myeloma. Subsequent positron emission tomography-computed tomography (PET-CT) scans showed widespread changes in bone structure throughout the body, particularly in the left scapular region, where there was notable thickening of surrounding soft tissue, subcortical osteolytic changes, and pathological fractures.

**Figure 1:**
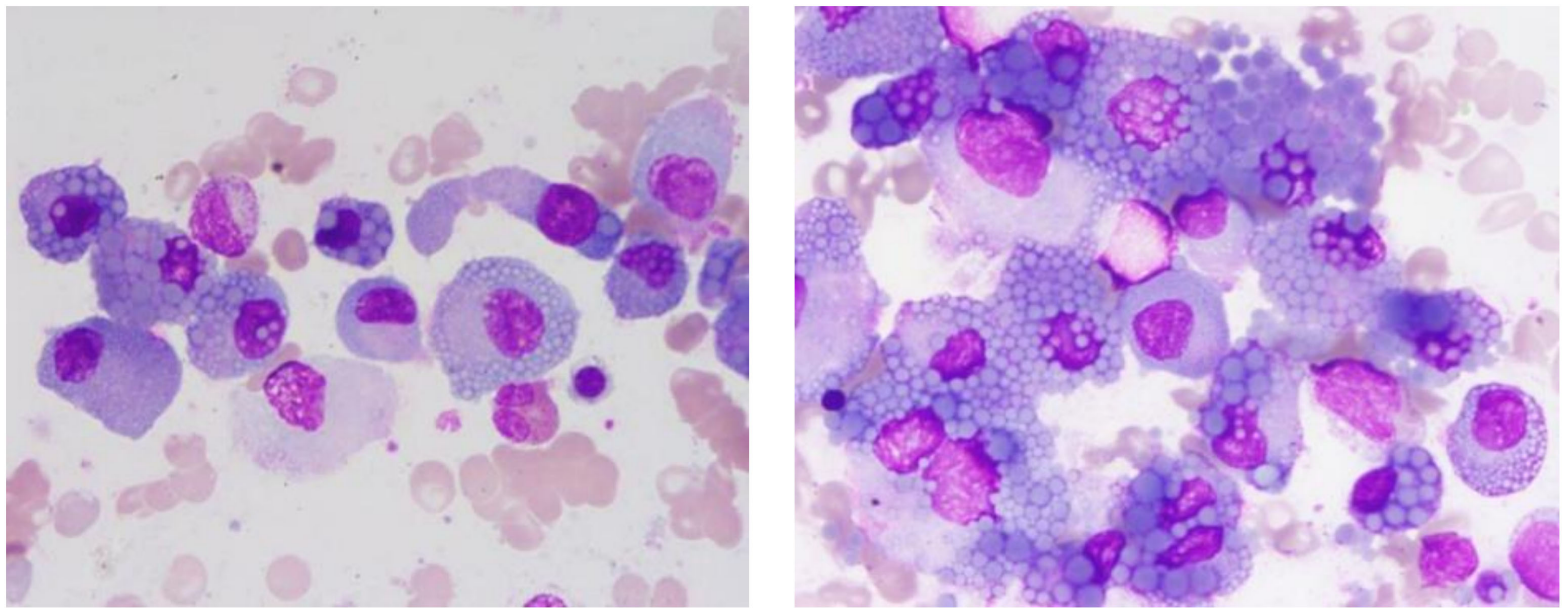
Abnormal plasma cell clusters in multiple myeloma. This bone marrow smear, stained with Wright-Giemsa and magnified 1000 times, displays a marked increase in abnormal plasma cells.

The patient’s complete blood count showed normal white blood cell, lymphocyte, and neutrophil counts, indicating no evident inflammatory or infectious response. However, reduced red blood cells and hemoglobin levels were observed, alongside a normal platelet count and elevated erythrocyte sedimentation rate (ESR). The reduction in red blood cells and hemoglobin indicated anemia, a common hematological presentation in patients with multiple myeloma. The normal platelet count excluded a bleeding tendency, and the significantly elevated ESR further suggested the presence of chronic inflammation or neoplastic activity. Kidney function tests showed elevated levels of urea, creatinine, cystatin C, and uric acid, reflecting impaired renal function, a common complication in multiple myeloma patients due to light chain deposition. Electrolyte levels (sodium, potassium, chlorine) were essentially normal, but levels of calcium, phosphorus, and magnesium were slightly elevated, likely related to bone destruction, another common clinical manifestation in these patients. Liver function tests revealed normal total protein but decreased albumin and increased globulin levels, leading to a reduced albumin/globulin ratio, indicative of poor nutritional status and a state of chronic inflammation. Elevated levels of β2-microglobulin were observed. Urinalysis revealed an increase in both white and red blood cells, with positive results for protein and nitrites, indicative of kidney damage and coagulation dysfunction. Coagulation tests showed elevated levels of D-dimer and fibrin degradation products, along with prolonged prothrombin time. Serum immunoglobulin IgG levels were elevated. Bone marrow cytology confirmed the diagnosis of multiple myeloma, with bone marrow smears revealing that myeloma cells accounted for up to 75% of the cellular makeup (**Figure 1**). These cells were larger, with round or near-round nuclei, often exhibiting nuclear displacement. The cytoplasm was filled with gray-blue foamy vacuoles, characteristic of Mott cells, which are highly correlated with the diagnosis of multiple myeloma.

**Figure 2:**
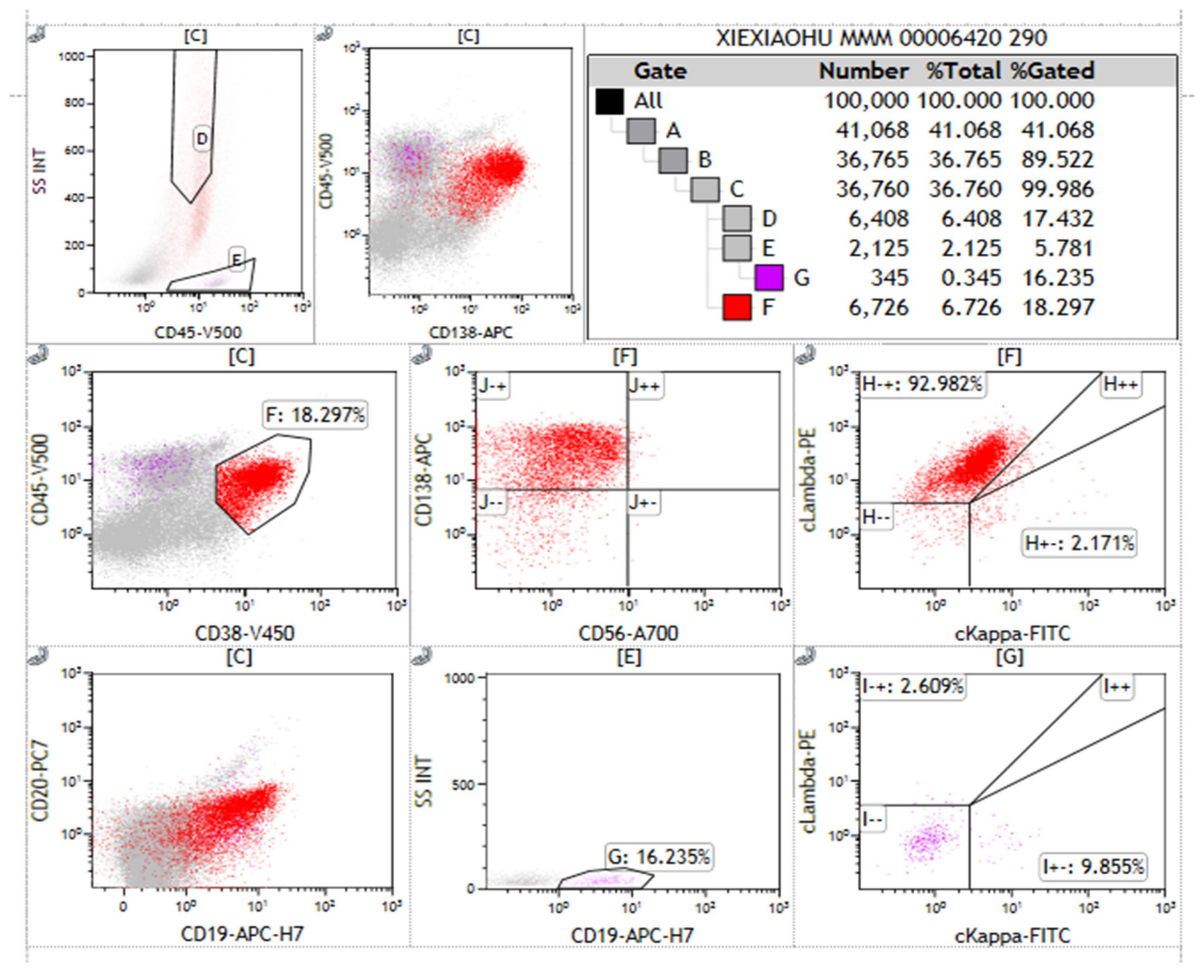
Immunophenotyping of abnormal plasma cells in multiple myeloma. This figure presents the flow cytometry immunophenotype analysis of abnormal plasma cells from a patient with Multiple Myeloma (MM) using a bone marrow aspirate sample separated into mononuclear cells via Ficoll. Each subplot represents different combinations of immune markers tested. Gates A to G correspond to different cell populations. Antibodies used in the flow cytometry analysis include CD45, CD38, CD138, CD19, CD56, CD20, cLambda, and cKappa light chains, with CD38 and CD138 identifying plasma cells. Of the 100,000 nucleated cells analyzed, 18.297% were identified as CD38+CD138+ abnormal plasma cells. These abnormal plasma cells showed positive expression for cLambda light chain and CD19, while not expressing cKappa light chain, CD56, or CD20. Statistical significance was assessed using standard percentage counting methods, with Gate F’s measurement serving as a representative analysis. Special markings: “-” indicates markers not detected; “+” indicates positive expression. The results exclude control samples.

**Figure 3:**
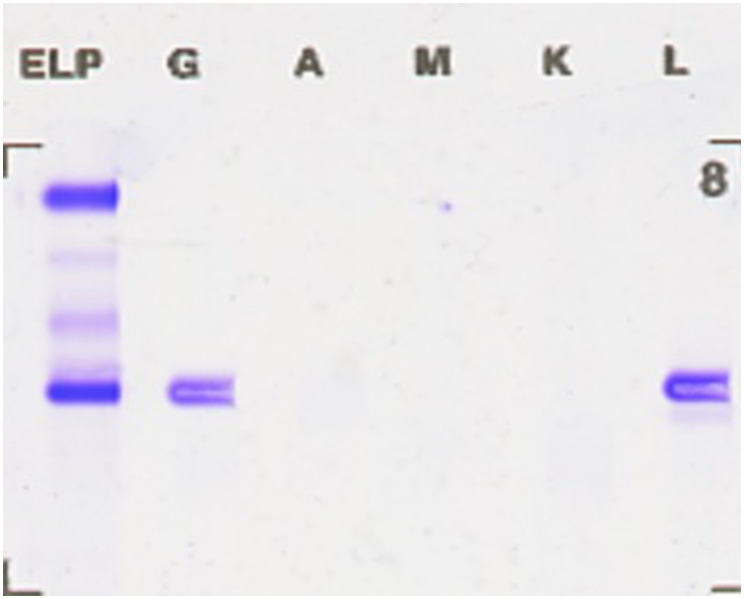
Serum immunofixation electrophoresis revealing IgG lambda bands. The bands located in the gamma region differ from the distribution of normal immunoglobulins, indicating an increase in monoclonal immunoglobulins, supporting the diagnosis of multiple myeloma.

Flow cytometry analysis of the immunophenotype of bone marrow cells showed that, after gating for CD45+ cells, lymphocytes accounted for 5.781% of nucleated cells, while abnormal plasma cells comprised 18.297%, displaying a phenotype positive for CD38, CD138, cLambda light chain, and CD19. These abnormal plasma cells did not express cKappa chains, CD56, or CD20, consistent with the common immunophenotype observed in patients with multiple myeloma (**Figure 2**). Serum immunofixation electrophoresis indicated an IgG lambda subtype, with bands located in the gamma region, differing from the distribution of normal immunoglobulins and indicating the presence of monoclonal immunoglobulins, supporting the diagnosis of multiple myeloma. The bands of IgG lambda proteins in the patient’s serum, corresponding with the bone marrow smear results, further confirmed the presence of multiple myeloma (**Figure 3**).

**Figure 4:**
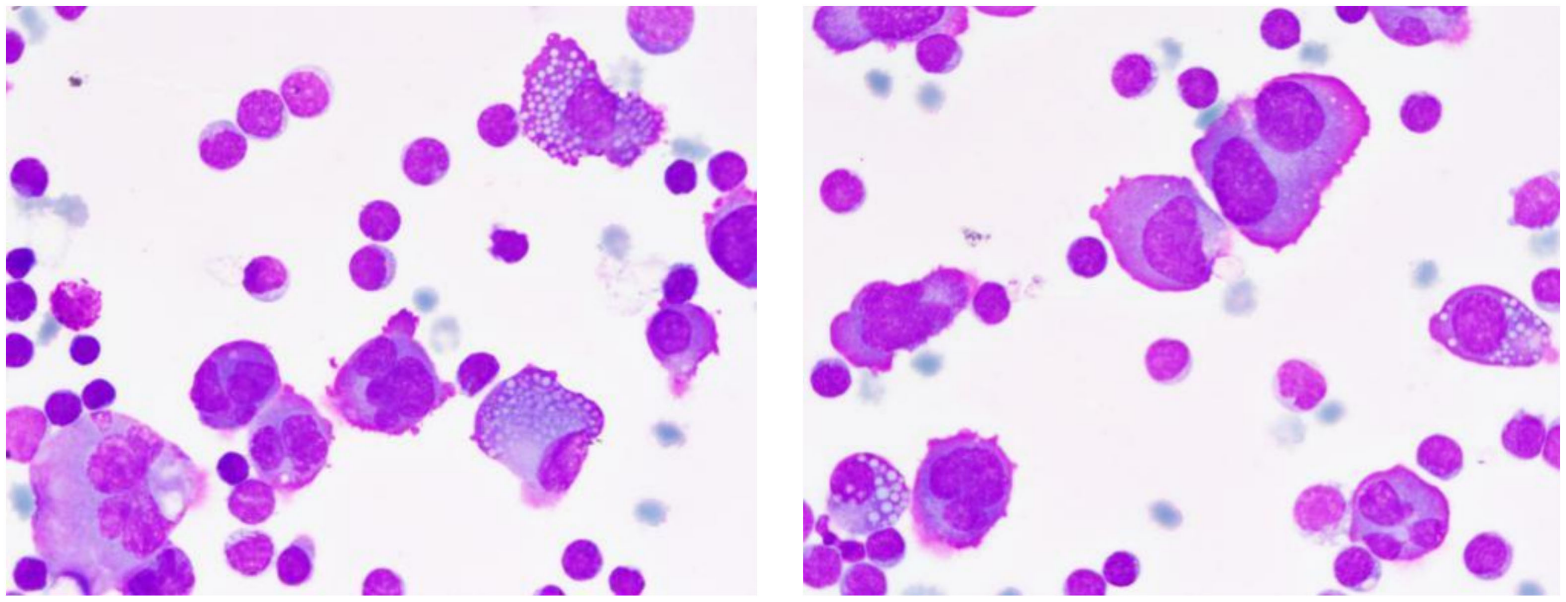
Characteristics of multiple myeloma cells in pleural effusion. This pleural fluid smear, stained with Wright-Giemsa and magnified 1000 times, showcases the typical morphological features of multiple myeloma cells. Cells vary in diameter from 20-40μm, with shapes ranging from round to irregular, rich cytoplasm with a purplish-red periphery and a bluish-purple perinuclear area, often containing numerous vacuoles. Nuclei frequently deviate from the center, appearing binucleated or irregularly shaped, with coarse and unevenly distributed chromatin. Arrows indicate Mott cells, a characteristic marker of multiple myeloma.

Urinary light chain analysis revealed a slight decrease in κ light chains and a significant increase in λ light chains, with blood light chain analysis showing a similar trend. Fluorescent *in situ* hybridization (FISH) revealed amplification of 1q21 and CMYC genes, deletion of 1p32, D13S319, and RB1 genes, and separation of the IGH gene. During the patient’s hospital treatment, left thoracic cavity drainage was performed, yielding yellow, turbid fluid without clots, and positive for Rivalta’s test, indicating significant increases in total cell and nucleated cell counts. Nucleated cell differentiation showed a predominance of lymphocytes, with macrophages and neutrophils comprising a minor portion and myeloma cells accounting for 20% (**Figure 4**).

The patient was admitted to the hospital on September 6, 2022, with a diagnosis of IgG lambda type multiple myeloma, staged as IIIb according to the Durie-Salmon staging system and stage III according to the International Staging System (ISS), accompanied by renal failure, severe hypoproteinemia, and malignant pleural effusion complications. In response to this complex condition, the PDD chemotherapy regimen was initiated on September 10, 2022, comprising bortezomib 1.9mg administered on days 1, 4, 8, and 11, dexamethasone 20mg given on days 1 to 4 and 8 to 11, and doxorubicin 40mg on day 6. Additionally, the patient received a variety of adjunctive treatments, including hydration and alkalinization therapy and zoledronic acid for bone resorption, bilateral thoracic cavity closed drainage, diuretic therapy, intravenous human albumin, blood transfusions, hemodialysis, anti-infection measures, and nutritional support, aimed at comprehensively improving the patient’s health status. Due to suboptimal control of the pleural effusion, on September 20, 2022, the physician decided to administer intrapleural injections of cyclophosphamide and dexamethasone, aiming for more effective management of the pleural effusion. Following this treatment, the patient’s left-side pleural effusion was reduced. To further evaluate the treatment’s efficacy, routine cytological examinations of the left and right pleural effusions were performed on September 28, 2022, revealing no myeloma cells. Additionally, flow cytometry analysis of the cellular immunophenotype showed no significant clusters of plasma cells in the pleural effusions on either side, indicating the treatment had achieved significant results, effectively controlling the malignant cells in the pleural effusion.

## Literature review

Multiple Myeloma (MM), a malignant tumor of the hematological system, is characterized by the accumulation of abnormally proliferating plasma cells in the bone marrow, leading to various clinical manifestations such as anemia, renal impairment, hypercalcemia, and bone lesions. MPE represents a rare but prognostically unfavorable clinical manifestation in patients with MM (17, 18). Its pathogenesis is not fully understood but may involve direct tumor invasion of the pleura, lymphatic obstruction, or protein-losing pleuritis. Early recognition and diagnosis are crucial for patient management and prognosis (19, 20). However, due to its nonspecific presentation and rarity, clinical diagnosis and treatment remain challenging. This paper underscores the novelty and importance of the diagnostic methods and treatment strategies discussed in this study by comparing them with previous research.

The detailed case report presented in this study showcases a rare instance of pleural effusion in a patient with multiple myeloma, revealing the etiology through a meticulous diagnostic process. Compared to previous literature, this study employed a comprehensive array of diagnostic approaches, including advanced imaging, laboratory testing, and immunophenotype analysis, to accurately identify the nature and source of the pleural effusion. This process not only demonstrates a systematic diagnostic strategy for complex cases but also highlights the importance of a multimodal approach in diagnosing rare conditions.

The treatment strategy for this case illustrates a comprehensive approach to managing multiple myeloma and its rare complications. Relative to previous studies, the treatment measures adopted here are more holistic, encompassing standard therapies for the primary disease, such as chemotherapy and targeted therapy, as well as specific interventions for complications, like intrapleural injections to manage pleural effusion. Moreover, this study emphasizes the significance of comprehensive patient management, including nutritional support, mental health, and symptom management, which are crucial for improving patient quality of life and treatment outcomes.

A review of recent literature indicates a significant correlation between the occurrence of MPE, increased tumor burden, and decreased survival rates (**Table 1**). Gao et al. (4), in a retrospective single-center study, noted that MPE patients had a heavier tumor burden and poorer prognosis compared to MM patients without this condition (4). Furthermore, Wang et al. (25) found that the use of invasive procedures to ascertain the exact cause of Pleural Effusion (PE) was suboptimal in a multicenter retrospective study (21). Kang et al. reviewed electronic medical records to identify risk factors for PE in MM patients, noting the significant incidence rate of PE in this population and its indication of poor prognosis (6).

**Table 1:** Summary table of literature on multiple myeloma with pleural effusion.

Journal	Authors	PMID	Year	Case Presentation	Diagnosis	Treatment	Outcome
Technol Cancer Res Treat	Gao L, Xu J, Xie W, et al.	36254566	2022	Retrospective Single-Center Study	Multiple Myeloma With Myelomatous Pleural Effusion	Assessed impact of myelomatous pleural effusion on prognosis	Myelomatous pleural effusion patients had heavier tumor burden and worse outcomes
Intern Med	Wang Z, Xia G, Lan L, et al. (25)	29217076	2016	Multicenter retrospective study	Multiple Myeloma with Pleural Effusion	Analyzed clinical features and practice patterns	Suboptimal use of invasive procedures to determine the exact cause of PE
Int J Gen Med	Kang Y, Hou ZL, Yang GZ, et al.	33658837	2021	Reviewed electronic medical records	Multiple Myeloma with Pleural Effusion	Investigated characteristics and identified risk factors for occurrence of PE in MM	Incidence of PE in MM patients is notable; PE indicates a poor prognosis
Blood	Touzeau C, Moreau P	26679866	2016	Review	Extramedullary myeloma	Described approach to management of EMM	Prognosis of EMM is poor, especially in patients with hematogenous EMM
Acta Haematol	Byun JM, Kim KH, Choi IS, et al.	28797003	2017	Multicenter retrospective study	Multiple Myeloma with Pleural Effusion	Analyzed clinical features and practice patterns	Real-world analyses showed suboptimal use of invasive procedures
Clin Lymphoma Myeloma Leuk	Yanamandra U, Deo P, Sahu KK, et al.	30704934	2019	Single-center Real-world Experience	Myelomatous Pleural Effusion	Systematically studied the incidence and clinicopathologic profile	MPE is rare and prognosis is dismal

In addressing Extramedullary Myeloma (EMM), Touzeau and Moreau (22) described approaches to managing EMM, emphasizing the particularly poor prognosis for patients with hematogenous EMM (22). Byun et al. (23) analyzed the clinical characteristics and practice patterns of MPE in a multicenter retrospective study, again highlighting the underuse of invasive procedures (23). Lastly, Yanamandra et al. (24) systematically studied the incidence and clinicopathological features of MPE through a single-center experience, confirming the rarity and grim prognosis of MPE (24).

These studies underscore the importance of identifying and managing PE in patients with MM. Despite limitations in the application of invasive diagnostic procedures, they play a crucial role in determining the exact cause of PE. Furthermore, given the poor prognosis associated with MPE, this patient group warrants further attention and research to improve treatment outcomes.

## Discussion

Multiple Myeloma (MM) is a malignancy of the hematological system, principally characterized by the aberrant proliferation of plasma cells in the bone marrow. These proliferating plasma cells produce large quantities of monoclonal immunoglobulins, leading to a spectrum of clinical manifestations, including anemia, renal impairment, hypercalcemia, and bone lesions. MPE represents a rare and usually prognostically adverse manifestation in patients with MM (17, 18). The precise pathogenesis of MPE remains unclear but is thought to involve multiple factors, such as direct tumor invasion of the pleura, lymphatic obstruction, or protein-losing pleuritis (19, 20), making early recognition and accurate diagnosis crucial for treatment selection and prognostic evaluation in affected patients.

In MM, the uncontrolled proliferation of plasma cells and the resultant production of monoclonal immunoglobulins are the primary culprits behind the functional impairment of related organs. While MM is typically confined to the bone marrow, a minority of patients may develop extramedullary myeloma (EMM), which can occur through several mechanisms (22): direct invasion of adjacent soft tissue by myeloma cells eroding the bone cortex, hematogenous dissemination to distant organs, or as a consequence of invasive surgical procedures or pathological fractures, affecting various sites including the skin, liver, pleura, and central nervous system.

The incidence of pleural effusion in patients with MM varies widely in the literature (4, 25), with potential causes ranging from chronic heart failure and renal failure to hypoalbuminemia, cirrhosis, and lymphatic obstruction. Notably, when MM directly invades the pleura, it results in what is termed MPE, typically signifying disease progression or relapse and indicating a poor prognosis (23, 24).

In this case, the patient was diagnosed with MM concurrent with the discovery of pleural effusion, highlighting the disease’s severity and the urgency of treatment. Through integrated therapeutic measures, including systemic chemotherapy combined with intrapleural injection treatment, tumor progression was successfully controlled. However, the patient’s renal function significantly worsened, accompanied by multiple complications. Ultimately, due to the rapid deterioration of the patient’s condition, including gastrointestinal perforation and hemorrhagic shock, the family opted to transfer him to a local hospital for further treatment. This case underscores the complexity and challenges of treating MPE while also emphasizing the importance of further research into this rare phenomenon to improve patient outcomes and quality of life.Moreover, this case also demonstrates that beyond standard chemotherapy, combining intrapleural injection, nutritional support, and other supportive interventions can effectively control MPE progression. This underlines the necessity of individualized treatment strategies tailored to each patient’s clinical condition.

The diagnosis of MPE encompasses four methods as reported in the literature (5, 24, 26, 27): (1) the detection of myeloma cells through cytological examination of pleural fluid; (2) identification of monoclonal protein components via electrophoresis of pleural effusion; (3) confirmation of myeloma cell invasion through pleural tissue biopsy or autopsy histological examination; and (4) detection of abnormal plasma cells in pleural effusion through Flow Cytometry (FCM). In this case, cytological analysis revealed 20% myeloma cells in the pleural effusion, affirming the diagnosis of MPE. Previous evidence (28) have shown that routine cytological screening of effusion for malignant cells offers high specificity. Routine cytological morphology examination of effusions, conducted by medical laboratories, involves centrifuging the effusion to obtain sediment for smear preparation, staining with Wright-Giemsa, and utilizing microscopy to screen for abnormal cells and perform cell type counts, assisting in clinical determination of effusion characteristics.

Reviewing recent literature reveals a significant correlation between the occurrence of MPE, increased tumor burden, and reduced survival rates (**Table 1**). Gao et al. (4), in a retrospective single-center study, highlighted that MPE patients exhibited a heavier tumor burden and worse prognosis compared to MM patients without this condition (4). Moreover, Wang et al. (25) found that the use of invasive procedures to ascertain the precise cause of Pleural Effusion (PE) was suboptimal in a multicenter retrospective study (21). Kang et al.’s study analyzed electronic medical records to identify risk factors for PE in MM patients, noting the significant incidence rate of PE in this population and its implication for poor prognosis (6).

In managing Extramedullary Myeloma (EMM), Touzeau and Moreau (22) described approaches to managing EMM, emphasizing the particularly poor prognosis for patients with hematogenous EMM (22). Byun et al. (23), in a multicenter retrospective study, analyzed the clinical characteristics and practice patterns of MPE, again highlighting the underuse of invasive procedures (23). Lastly, Yanamandra et al. (24) systematically studied the incidence and clinicopathological features of MPE through single-center experience, confirming the rarity and grim prognosis of MPE (24).

These studies underscore the importance of identifying and managing PE in patients with MM. Despite limitations in the application of invasive diagnostic procedures, they play a crucial role in determining the exact cause of PE. Furthermore, given the poor prognosis associated with MPE, this patient group requires additional attention and in-depth research to improve treatment outcomes. To address this, integrating cytological examination, immunophenotypic profiling, and genetic analysis proves essential for accurate diagnosis, risk stratification, and the formulation of personalized treatment strategies.

## Conclusion

This study meticulously documents and analyzes the comprehensive diagnostic and therapeutic journey of a 73-year-old male patient with multiple myeloma, showcasing effective management strategies for the complex complication of MPE. Utilizing a range of diagnostic tests, including hematology, biochemistry, imaging, and molecular genetics, alongside a personalized, integrated treatment plan encompassing PDD chemotherapy, adjuvant pharmacotherapy, and closed thoracic drainage, the study not only successfully managed the patient’s pleural effusion but also significantly improved his overall health status, alleviating symptoms and enhancing the quality of life. Furthermore, this research contributes significantly to understanding the complexity of multiple myeloma, improving the accuracy of diagnosing its complications, and developing more effective treatment protocols, offering valuable insights for the treatment of similar cases in the future. Furthermore, the success of this case reflects the importance of integrating multiple factors—such as complications, molecular features, and overall health status—into personalized care plans. The use of PDD chemotherapy combined with pleural interventions and supportive therapies offers a promising model for managing similarly complex clinical scenarios.

**Figure 5:**
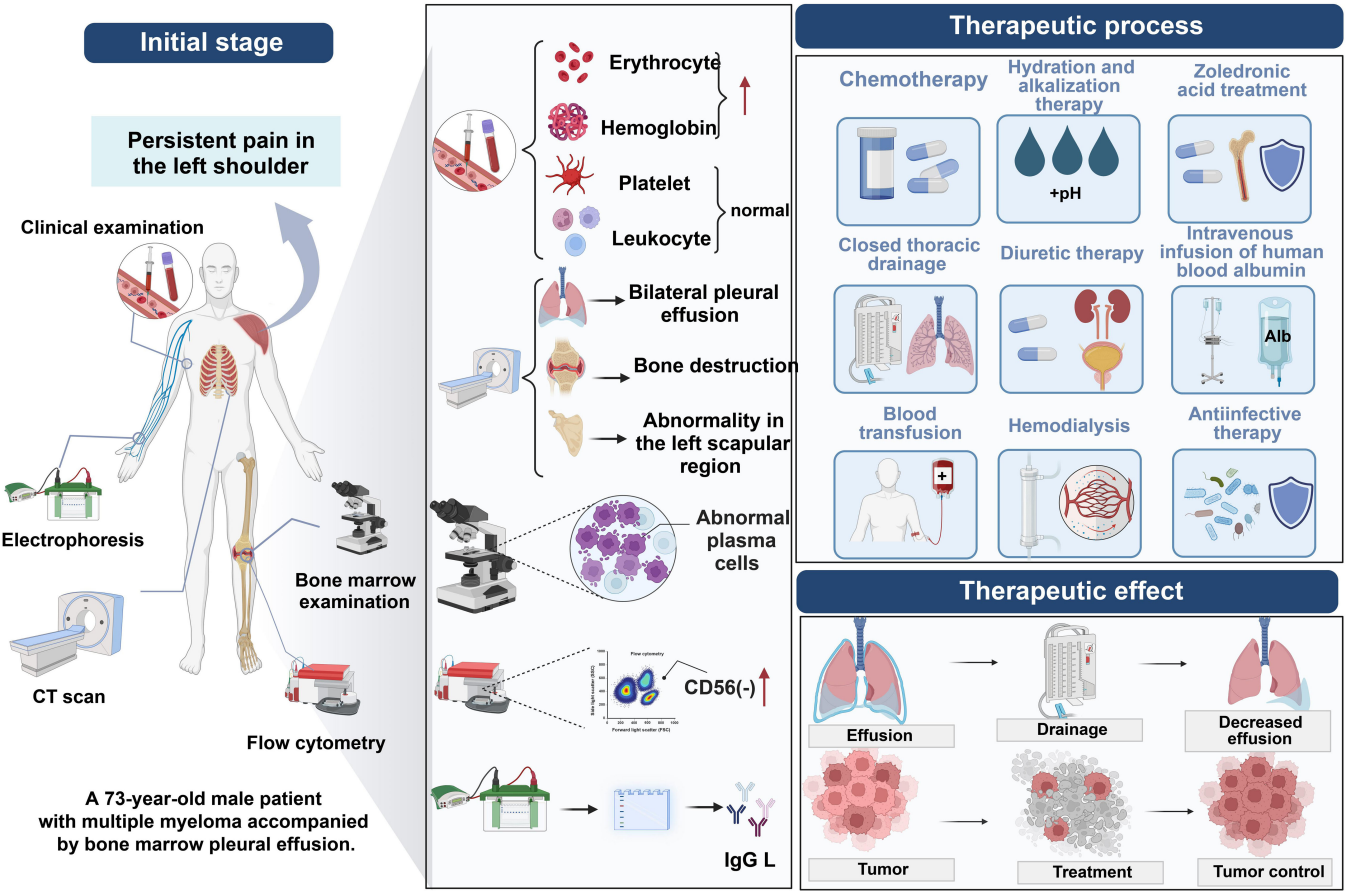
Diagnostic process for myelomatous pleural effusion in multiple myeloma using cytological analysis of pleural fluid.

Routine cytological examination of pleural effusion emerges as an economical, convenient, fast, and highly specific method poised to play an increasingly important role in enhancing the detection rate of malignant pleural effusion (MPE) in future research and clinical practice. The advantage of this method lies not only in its simplicity and cost-effectiveness but also in providing a crucial basis for stratified diagnosis and treatment of pleural effusion caused by multiple myeloma and other diseases. Looking ahead, further optimization of cytological analysis techniques for pleural effusion, combined with advanced molecular biological methods such as Fluorescent *In Situ* Hybridization (FISH) and flow cytometry, will enable more precise identification and classification of malignant cells in pleural effusion. This will not only deepen understanding of diseases like multiple myeloma but also advance personalized medicine, offering patients more accurate treatment plans and ultimately improving survival rates and quality of life. Additionally, applying these methods in clinical practice will help establish a more comprehensive and systematic diagnostic and therapeutic process, providing patients with continuous medical services from precise diagnosis to personalized treatment (**Figure 5**).

## Data Availability

The original contributions presented in the study are included in the article/supplementary material. Further inquiries can be directed to the corresponding author.
